# Effects of gamma irradiation on cell cycle, apoptosis and telomerase activity in p53 wild-type and deficient HCT116 colon cancer cell lines

**DOI:** 10.3892/ol.2013.1441

**Published:** 2013-07-03

**Authors:** SEVIL OSKAY HALACLI, HANDE CANPINAR, EREN CIMEN, ASUMAN SUNGUROGLU

**Affiliations:** 1 Pediatric Immunology Unit, Institute of Children's Health, Hacettepe University, Sihhiye, Ankara 06100, Turkey; 2 Department of Basic Oncology, Institute of Oncology, Hacettepe University, Sihhiye, Ankara 06100, Turkey; 3 Pediatric Hematology Unit, Institute of Children's Health, Hacettepe University, Sihhiye, Ankara 06100, Turkey; 4 Department of Medical Biology, Faculty of Medicine, Ankara University, Sihhiye, Ankara 06100, Turkey

**Keywords:** irradiation, cell proliferation, p53, apoptosis, telomerase activity

## Abstract

Radiotherapy serves as adjunctive treatment to chemotherapy and surgical resection of colorectal cancer. However, the cellular response to irradiation varies depending on the expression of tumor suppressor p53, which plays a significant role in the regulation of cell cycle arrest, apoptosis and telomerase activity in various cancers. The present study aimed to investigate cell cycle arrest, apoptosis and telomerase activity with respect to p53 expression in p53 wild-type (+/+) and deficient (−/−) HCT116 colon cancer cell lines following 5 Gy γ-irradiation. Cell cycle arrest and apoptosis were evaluated using flow cytometry. The telomerase activity was measured using a TRAP (telomerase repeat amplification protocol) assay. Following treatment with irradiation, G_1_/S cell cycle arrest occurred in the p53+/+ cells, whereas the p53−/− cells accumulated in the G_2_ phase. No differences were observed in the apoptotic ratios between the two cell lines following irradiation. Decreased telomerase activity was observed in the p53+/+ cells, whereas telomerase activity was increased in the p53−/− cells. The results showed that while telomerase activity and G_1_ cell cycle arrest were regulated depending on the p53 status, G_2_ arrest and the apoptotic response were promoted via a p53-independent pathway.

## Introduction

Colorectal cancer is one of the leading causes of cancer-associated mortality in the world. According to the linear model of cancer initiation, proposed by Fearon and Vogelstein, cancer is a disease that arises from multiple serial somatic mutations (1). The characteristic alterations to this model comprise mutations of the tumor suppressor genes, including APC, TP53 and K-Ras. TP53 mutations have been identified in >50% of human tumors. Since TP53 is a tumor suppressor gene, loss of function mutations are the general cause of abnormality (1).

The TP53 gene is known as the guardian of the genome or the cellular gatekeeper. The gene contains 11 exons, which encode 2.8 kb mRNA that is translated into a 53 kDa protein. Following exposure to stress conditions, including hypoxia, oncogene activation, DNA damage, nucleotide defects and viral transformation, p53 is subjected to certain post-translational modifications that regulate the subcellular localization and stability of the protein (2–5). Under these stress conditions, there are three cellular outcomes: i) Repair mechanisms are prompted (5); ii) if there is no way to avoid it, the cells undergo apoptosis or cell cycle arrest (6–17); and iii) the cells defense mechanisms are affected and the cells become cancerous. p53 may act as a key downstream regulator for all the processes mentioned previously and also for telomerase activity. p53 regulates the inactivation of the catalytic subunit of telomerase, which is a reverse transcriptase that is activated in cancer cells (18,19). However, there are questions that require clarification with regard to cell fate, since there is no clear mechanism or requirement as to which possible outcome is preferred. In the present study, the following two issues were examined: i) The preferred mechanism of cell fate following 5 Gy γ-irradiation, depending on p53 expression in p53 +/+ and p53 −/− HCT116 colon cancer cells; and ii) whether the cell response is p53-associated or p53-independent in HCT116 colon cancer cells.

## Materials and methods

### Cell culture and irradiation

p53+/+ and p53−/− HCT116 colon cancer cell lines were cultured in complete McCoy's 5A medium, consisting of 10% fetal bovine serum, 1% penicillin/streptomycin and 1% L-Glutamine at 37°C, in a humidified incubator containing 5% CO_2_. Once 80–90% confluency was reached in T75 culture flasks, the cells were treated with 5 Gy γ-irradiation (Co60-Dmax) and collected to evaluate the cell cycle, apoptosis and telomerase activity.

### Detection of apoptotic cells and analysis of the cell cycle

Following irradiation, the trypsinized cells were washed and collected by centrifugation. The cell numbers were counted using a hemocytometer. RNase (Sigma, St. Louis, MO, USA) and propidium iodide (Sigma) were added to the cells and mixed using a vortex. Following a 20-min incubation period in the dark at room temperature, the cells were filtered through a nylon mesh (37 μm) and evaluated using flow cytometry (EPICS XL MCL; Beckman Coulter Inc., Brea, CA, USA). The ratios of the cells in the G_0_/G_1_, S and/or G_2_/M phases and the apoptotic cell numbers were evaluated by McCycle software (Phoenix Flow System, San Diego, CA, USA) using dichotomous variable DNA histograms.

### Telomerase activity

Protein lysates were prepared from a CHAPS lysis buffer and quantified using the Bradford method. In order to detect telomerase activity, the TRAPeze XL Telomerase Detection kit (Chemicon, Temecula, CA, USA) was used.

### Statistical analysis

The statistical analyses were performed using SPSS software version 13 (SPSS, Inc., Chicago, IL, USA). The variables were investigated using visual (histograms and probability plots) and analytical (Kolmogorov-Simirnov/Shapiro-Wilk's test) methods to determine whether or not they were normally distributed. The χ^2^ test was used to statistically analyze the cell cycle and apoptosis. The telomerase activity was evaluated using a Mann-Whitney U test. P<0.05 was considered to indicate a statistically significant difference.

## Results

### Apoptosis

**Figure 1:**
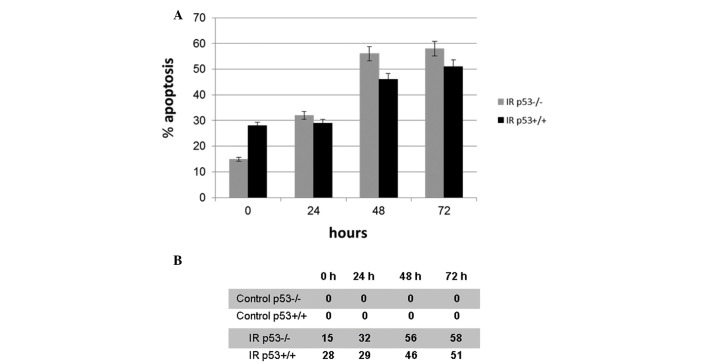
(A) Average apoptotic percentages obtained from three independent experiments in irradiated and non-irradiated p53 wild-type (+/+) and deficient (−/−) HCT116 cells. (B) The average apoptotic cell numbers of irradiated and non-irradiated control cells. Values of <10% were considered to be the threshold in the irradiated control p53−/− and p53 +/+ cells. IR, irradiated.

The apoptotic ratios of the cells following exposure to irradiation were evaluated using the sub-G_0_ DNA content. Following treatment with 5 Gy γ-irradiation, the average apoptotic percentages of cell number significantly increased in a time-dependent manner in the p53+/+ and p53−/− cells (P<0.05), whereas there was no change in the apoptotic cell number in the non-irradiated control cells (Fig. 1A). The average apoptotic cell numbers of the irradiated and non-irradiated control cells are demonstrated in Fig. 1B. Values of <10% were considered to be the threshold in the non-irradiated control p53−/− and p53+/+ cells.

### Cell cycle analysis

**Figure 2:**
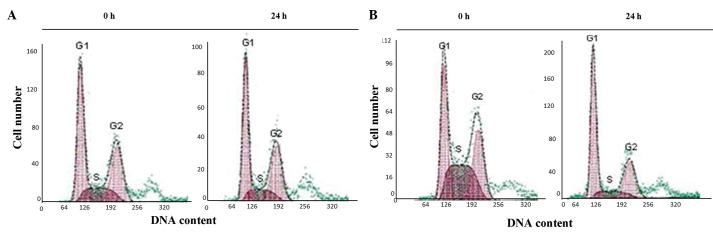
Normal distribution of G_1_, S and G_2_ stages of the cell cycle in non-irradiated (A) p53 wild-type (+/+) and (B) p53 deficient (−/−) HCT116 cells at 0 and 24 h.

**Figure 3:**
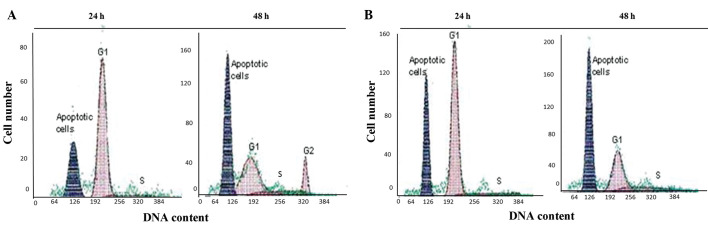
(A) G_1_ phase arrest at 24 h and accumulation in G_2_ phase arrest at 48 h in irradiated p53 deficient (−/−) HCT116 cells. (B) G_1_ and S phase arrest in irradiated p53 wild-type (+/+) HCT116 cells at 24 and 48 h. Blue areas of the histogram illustrate apoptotic cells associated with the sub-G_0_ DNA content.

The G_1_, S and G_2_ phases of the cell cycle showed a normal distribution in the non-irradiated p53+/+ and p53−/− control cells (Fig. 2). However, following exposure to irradiation, the p53+/+ cells became permanently accumulated in the G_1_ phase within 24 h. At 48 h post-irradiation, the cells passed to the S phase and arrested there. The apoptotic cell numbers, according to the sub-G0 DNA contents, were increased at the indicated time-points in the p53+/+ cells following irradiation. (Fig. 3). The irradiated p53−/− cells also showed a similar pattern to the p53+/+ cells within 24 h. However, the cells that escaped from the G_1_ phase accumulated in the G_2_ phase at 48 h (Fig. 3). Overall, the apoptotic cell numbers of the irradiated p53−/−cells showed similar patterns to the irradiated p53+/+ cells.

### Telomerase activity

**Figure 4:**
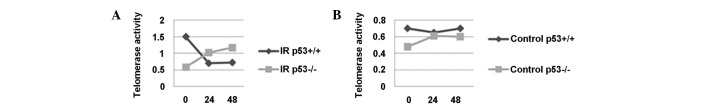
Comparison of telomerase activity in p53 wild-type (+/+) and p53 deficient (−/−) cells in (A) irradiated and (B) non-irradiated cells. IR, irradiated.

The telomerase activity of the non-irradiated p53+/+ cells was nearly uniform at 0, 24 and 48 h. The telomerase activity of the non-irradiated p53−/− cells was marginally different to the p53+/+ cells at the designated time-points. In the irradiated p53+/+ cells, the telomerase activity was low at 0 h and continued to decrease at 24 and 48 h. In contrast to this, the telomerase activity was similar to the non-irradiated p53−/− cells at 0 h. However, at 24 h post-irradiation, the telomerase activity increased and remained at a higher level at 48 h (Fig. 4).

## Discussion

γ-irradiation causes double or single strand breaks depending on the application dose, for example 5 or 7.5 Gy, in cells (20). Following single or double strand DNA breaks, time protective processes should be activated (16). The four checkpoints during the cell cycle are at G_1_/S, S, G_2_/M and M. If there is a problem in any of the checkpoints, the cycle is stopped and allowed sufficient time for repair. However, in certain cases, the repair pathways themselves may be defective due to abnormal enzyme activity in the signaling cascade. p53 prevents cell proliferation via telomere shortening and telomerase activity and by activating cellular senescence. In this case, an alternative conserving mechanism, which is generally termed apoptosis, is activated. Therefore, cells are protected from transferring the wrong copies to their daughter cells. The cellular gatekeeper p53 protein is situated in the nucleus and activates genes that are responsible for repair, apoptosis and telomere regulation (6–7,11,21). The present study aimed to clarify the p53 dependency of G_1_ and G_2_ arrest, apoptosis and telomerase activity following 5 Gy γ-irradiation in p53+/+ and p53−/− HCT116 colon carcinoma cells.

In the present study, the HCT116 cells expressing p53+/+ were arrested in the G_1_ phase of the cell cycle at 24 h and at 48 h, then escaped from G_1_ arrest and accumulated in the S phase, driving apoptosis at an additional 48 h after 5 Gy γ-irradiation. Attardi *et al* showed that p53+/+ MEF (mouse embryonic fibroblast) cells accumulated in G_1_ phase arrest following 5 Gy irradiation. Following irradiation, the p21 promoter is triggered depending on the increased expression of p53 in the MEFs. However, p53−/− MEFs underwent G_2_ phase arrest subsequent to irradiation. G_2_ arrest in the MEFs occurred independently of p53 (12). There is evidence from certain studies that suggests that the CDC25A molecule is significant in p53-independent G_2_ phase arrest (22). CDC25A is one of the key molecules involved in the cell cycle, and following treatment with γ-irradiation, CDC25A is decreased in cells. Thus, the cells are prevented from entering the M phase by a p53-independent mechanism. p21+/+ and p53−/− HCT116 cells undergo G_2_ phase arrest instead of G_1_ phase arrest following irradiation. In the p53−/− cells of the present study, p21 and CDK may have regulated p53-independent G_2_ arrest. The G_1_ and G_2_ arrests were accompanied by cell death with an increasing rate of occurrence of ≤2 h after exposure in the p53+/+ and p53−/− cells. According to a study using *Drosophila melanogaster* as a model, following ionizing radiation treatment, apoptosis occurred in a p53-independent manner (23).

While telomerase activity was decreased in the p53−/− cells of the present study, increased telomerase activity was identified in the p53+/+ cells following γ-irradiation. The activity of TERT, which is a catalytic subunit of telomerase, depends on whether p53 is expressed. Following irradiation, TERT activity is decreased depending upon the level of p53 expression. However, in p53−/− cells, TERT activity is increased due to the absence of p53 (24). There are various studies with regard to the association between p53 and telomerase activity, and a number of conclusions have been made. In a study using pituitary adenoma cells with no p53 expression, telomerase activity was increased during malignant transformation (25). While 10 Gy γ-irradiation has not been shown to alter telomerase activity, accelerated senescence has been observed in p53+/+ MCF7 breast cancer cells (26). Subsequent to 0.1–1 Gy doses of X-rays, telomerase activity and telomere lengthening were induced in TK6-expressing p53+/+ and p53 mutant WTK1 human lymphoblast cell lines. However, the triggering of telomerase activity following radiation was not believed to be associated with the p53 pathway (21).

In summary, the exposure to 5 Gy γ-irradiation, telomerase activity and G_1_ cell cycle arrest were regulated depending on the p53 status in the HCT116 colon cancer cells. However, G_2_ arrest and the apoptotic response were promoted in a p53-independent pathway.
